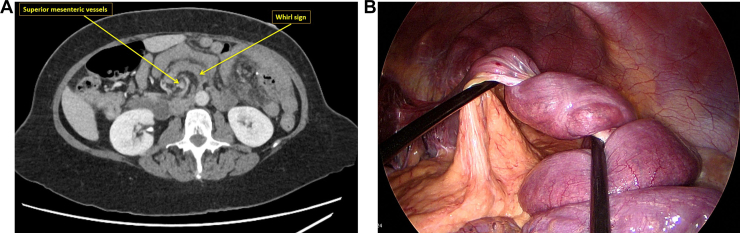# A 360-Degree Alimentary Limb Volvulus After Roux-en-Y Gastric Bypass

**DOI:** 10.1016/j.gastha.2026.100962

**Published:** 2026-04-10

**Authors:** Christian Mouawad, Abdessalem Ghedira, Nehad Dager

**Affiliations:** Department of Digestive Surgery, Centre Hospitalier Intercommunal de Villeneuve-Saint-Georges, Villeneuve-Saint-Georges, France

A 56-year-old woman with prior laparoscopic sleeve gastrectomy converted to Roux-en-Y gastric bypass presented with 1 week of progressively worsening diffuse abdominal pain. Physical examination showed diffuse abdominal tenderness with left-sided guarding, while laboratory tests were unremarkable. Contrast-enhanced computed tomography revealed a whirl sign of the mesenteric root with twisting around the superior mesenteric vessels and marked mesenteric edema, without pneumatosis or free air (Figure A).

Emergency surgical exploration demonstrated milky intraperitoneal fluid and a striking 360-degree volvulus of the alimentary limb around its mesenteric axis (Figure B). The intraoperative image clearly shows the alimentary limb spiraled tightly on itself, with torsion of the mesentery and congested, violaceous bowel loops, illustrating the complete rotational nature of the volvulus. The volvulus resulted from herniation of the small bowel through a wide Petersen’s defect, with associated venous congestion and mesenteric engorgement.

After unsuccessful laparoscopic reduction, conversion to laparotomy allowed complete devolvulation, with rapid restoration of bowel color and viability. Both Petersen’s defect and the jejunojejunal mesenteric defect were closed. Postoperatively, bowel function resumed promptly, and the patient was discharged on postoperative day 5 without complications.

**Figure:**